# Evaluation of augmented reality training for a navigation device used for CT-guided needle placement

**DOI:** 10.1007/s11548-024-03112-3

**Published:** 2024-05-08

**Authors:** T. Stauffer, Q. Lohmeyer, S. Melamed, A. Uhde, R. Hostettler, S. Wetzel, M. Meboldt

**Affiliations:** 1 Product Development Group Zurich, ETH Zurich, Leonhardstrasse 21, 8092 Zurich, Switzerland; 2 Medical Templates AG, Technoparkstrasse 1, 8005 Zurich, Switzerland; 3 Department of Neuroradiology, Hirslanden Clinic Zurich, Witellikerstrasse 40, 8032 Zurich, Switzerland

**Keywords:** Medical education, Augmented reality, User study, Interventional radiology

## Abstract

**Purpose:**

Numerous navigation devices for percutaneous, CT-guided interventions exist and are, due to their advantages, increasingly integrated into the clinical workflow. However, effective training methods to ensure safe usage are still lacking. This study compares the potential of an augmented reality (AR) training application with conventional instructions for the Cube Navigation System (CNS), hypothesizing enhanced training with AR, leading to safer clinical usage.

**Methods:**

An AR-tablet app was developed to train users puncturing with CNS. In a study, 34 medical students were divided into two groups: One trained with the AR-app, while the other used conventional instructions. After training, each participant executed 6 punctures on a phantom (204 in total) following a standardized protocol to identify and measure two potential CNS procedural user errors: (1) missing the coordinates specified and (2) altering the needle trajectory during puncture. Training performance based on train time and occurrence of procedural errors, as well as scores of User Experience Questionnaire (UEQ) for both groups, was compared.

**Results:**

Training duration was similar between the groups. However, the AR-trained participants showed a 55.1% reduced frequency of the first procedural error (*p* > 0.05) and a 35.1% reduced extent of the second procedural error (*p* < 0.01) compared to the conventionally trained participants. UEQ scores favored the AR-training in five of six categories (*p* < 0.05).

**Conclusion:**

The AR-app enhanced training performance and user experience over traditional methods. This suggests the potential of AR-training for navigation devices like the CNS, potentially increasing their safety, ultimately improving outcomes in percutaneous needle placements.

## Introduction

**Fig. 1:**
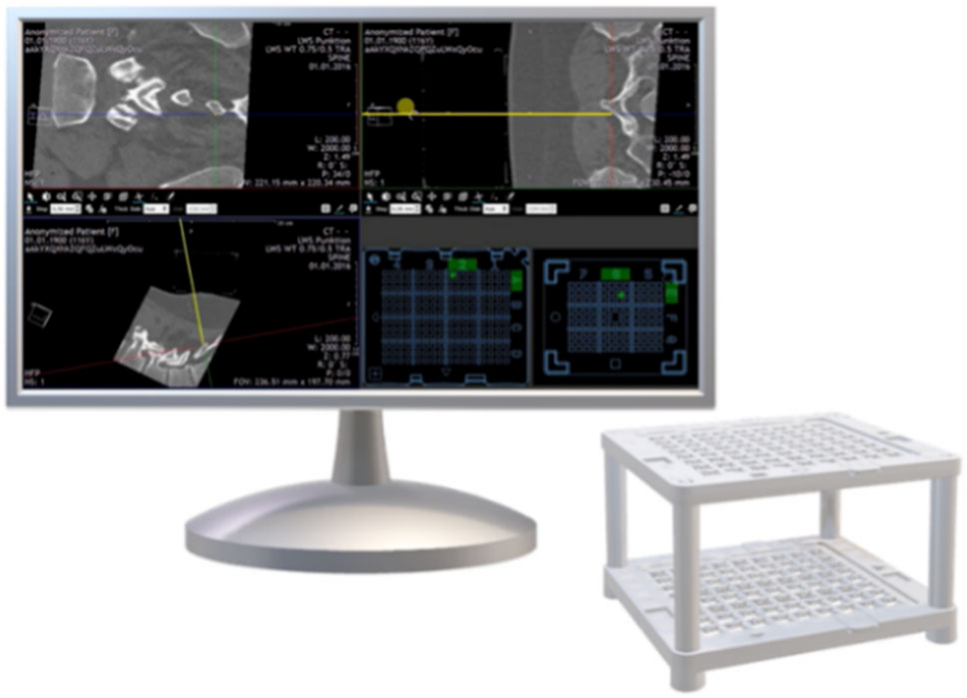
Medical Templates planning software and Navigation Cube. The yellow line visible in CT-scans represents a virtual needle which is used to plan the puncture. As the needle moves, a green dot representing the coordinates in the top and bottom plates (shown in the bottom right quadrant) automatically updates. The required angle is achieved by inserting the needle through the corresponding holes and corners on the top and bottom plates of the Navigation Cube

Percutaneous CT-guided interventions are widely employed in the medical field, including pain therapies, biopsies, and ablations [1, 2]. Precise placement of the needle is crucial for the success of these procedures, as it helps prevent tissue damage, provides accurate diagnostic results, and leads to effective treatment outcomes [1]. With the advent of navigation software and robotic assistance, needle placement has become safer and more efficient in recent years [3, 4]. One promising technique is the utilization of patient-mounted devices, such as Navigation Cubes, being part of the Cube Navigation System (CNS) (see Fig. 1). This method has been demonstrated to improve accuracy and reduce intervention time when compared to the traditional freehand method (FHM) [5, 6].

Due to these advantages, the clinical use of the CNS is increasing. However, as adoption grows, effective training methods become important to ensure safe usage of the system. So far, training included written instructions, which are solely text-based and therefore detached from the application. This complicates the information processing for learners, as no hands-on context is provided. Moreover, written instructions lack a mechanism to verify users' comprehension of the system.

To find a possible solution addressing these issues, the present study evaluates the effectiveness of augmented reality (AR) training in teaching radiologists the usage of the CNS. An AR-tablet app was specifically designed to ease the understanding of the application by providing the information gradually with a hands-on context and to enable immediate feedback as errors occur.

The aim of this study is to compare AR-training to conventional instruction when teaching radiologists puncturing with the CNS. The results of this study are expected to demonstrate the advantage of AR-training over conventional instruction in terms of patient safety for two main reasons: (1) It simplifies the presentation of information by providing it in a gradual and contextual manner. (2) It provides immediate feedback, allowing users to identify and correct errors. These advantages are supposed to contribute to the quicker and safer adoption of the CNS in medical practice compared to conventional written instructions, ultimately reducing the risk of tissue injury, incorrect diagnosis, and ineffective treatment outcomes.

### Related works

#### Augmented reality in medical education

AR has found a growing application in medical education, particularly in the learning of anatomy, diagnosis, procedural planning and execution, as well as medical device handling. It offers unique benefits, especially for learning new processes [7, 8].

Lia et al. developed an AR-based training module using HoloLens^®^ to teach basic suturing skills. The holographically displayed video instructions aimed to provide an interactive, self-directed learning experience. In a comparative study, AR-training was not shown to achieve better suturing results than conventional instructions, but users found the app to be generally helpful and engaging, indicating increased user acceptance [9].

Azimi et al. implemented an AR-instructor using HoloLens^®^ to train 20 novice caregivers on two emergency medical procedures: (1) needle chest decompression and (2) initiating a direct intravenous line. The study found that using AR-training was more engaging and improved time on task, as well as increased confidence in executing the procedures when compared to conventional instruction methods [10].

Wolf et al. evaluated the effectiveness of AR-based step-by-step guidance for extracorporeal membrane oxygenation (ECMO) cannulation training using the Microsoft HoloLens 2^®^. The study found that the use of AR-instructions resulted in a slight increase in training time but a significant reduction in errors, particularly knowledge-related errors, compared to conventional training methods [11].

By evaluating AR-training on the CNS, this study aims to further investigate the effectiveness of AR in medical education.

#### Training of freehand method in image-guided needle placement

Several computer-aided simulators have been developed for the training of the FHM. These methods offer focused and deliberate practice with continuous feedback, essential for acquiring these types of intervention skills.

Ungi et al. presented an open-source platform for training in ultrasound-guided needle insertions called Perk Tutor. It replaces traditional patient training with computer-assisted guidance. Using electromagnetic tracking, it localizes needle, probe, and phantom for various procedures. The platform offers real-time feedback and records needle trajectories for enhanced learning [12].

Holden et al. used in their study the Perk Tutor platform for the training of image-guided needle placement and applied machine learning techniques for skills evaluation. Feedback from these automated methods was then benchmarked against expert opinions, showing comparable scores in most scenarios [13].

Hayasaka et al. trained in their study 30 medical students in needle placement using traditional methods and AR-techniques. The AR-enhanced groups, which utilized HoloLens 2^®^ for visualization, reported significantly better user experiences in the training. Overall, AR-training showed promise in enhancing the learning of epidural needle placement techniques [14].

In contrast to the training for the FHM, the authors are not aware of any study investigating training on navigation aids for percutaneous needle placements despite their growing clinical adoption. This study aims to fill this gap by developing an AR-training app for the CNS and evaluating its potential to ensure the safety of its usage in clinical practice.

## Methods

### Cube Navigation System

The Cube Navigation System (Medical Templates AG, Egg, Switzerland) (Fig. 1) is a navigation aid for CT-guided punctures, comprising planning software and a patient-mounted cube accessory. The Navigation Cube, visible in CT images, is placed on the patient directly over the target region of the puncture. Once a scan is taken, the software automatically localizes the cube. Then, the planned trajectory of the puncture is referenced to the top and bottom grids, determining the coordinates through which the needle must be inserted to achieve the desired needle trajectory. (A coordinate is defined as a particular corner in a hole of the grid.) Compared to the conventional FHM, the CNS thereby allows for a reduction in the time-consuming stepwise control of the needle trajectory with imaging techniques [15].

When puncturing with the Navigation Cube, there are two potential sources of user error leading to an inaccurate puncture: (1) using incorrect coordinates due to a slip in the column or row and (2) rotating the cube due to applying pressure during the puncture, causing a shift in the cube and puncture trajectory.

### AR-training app

The training app was built using the Unity^®^ 3D game engine (Unity Technologies, San Francisco, California), a platform for creating interactive applications. The Vuforia^®^ Augmented Reality SDK (PTC Inc., Boston, Massachusetts) was integrated into the Unity environment to facilitate the display of holograms and tracking of the Navigation Cube.

**Fig. 2:**
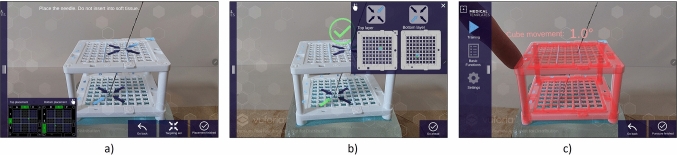
Snapshots of three major steps of AR-training: **a** display of coordinates for needle placement, ** b** user feedback after verification of needle’s correct position, **c** holographic warning to maintain minimal rotation during puncturing

The tablet app provides a training platform that guides the user gradually through the procedure of puncturing using the Navigation Cube and verifies each step's correct execution. The training process is divided into three major steps (Fig. 2): (1) needle placement, (2) verification of the needle's correct position, (3) performing the puncture.

First, an image from the planning software is shown to the user. This image illustrates the coordinates where the needle needs to be placed on the Navigation Cube for the puncture. If desired, the user can activate an AR-hologram indicating the exact coordinate by a light blue arrow.

**Fig. 3:**
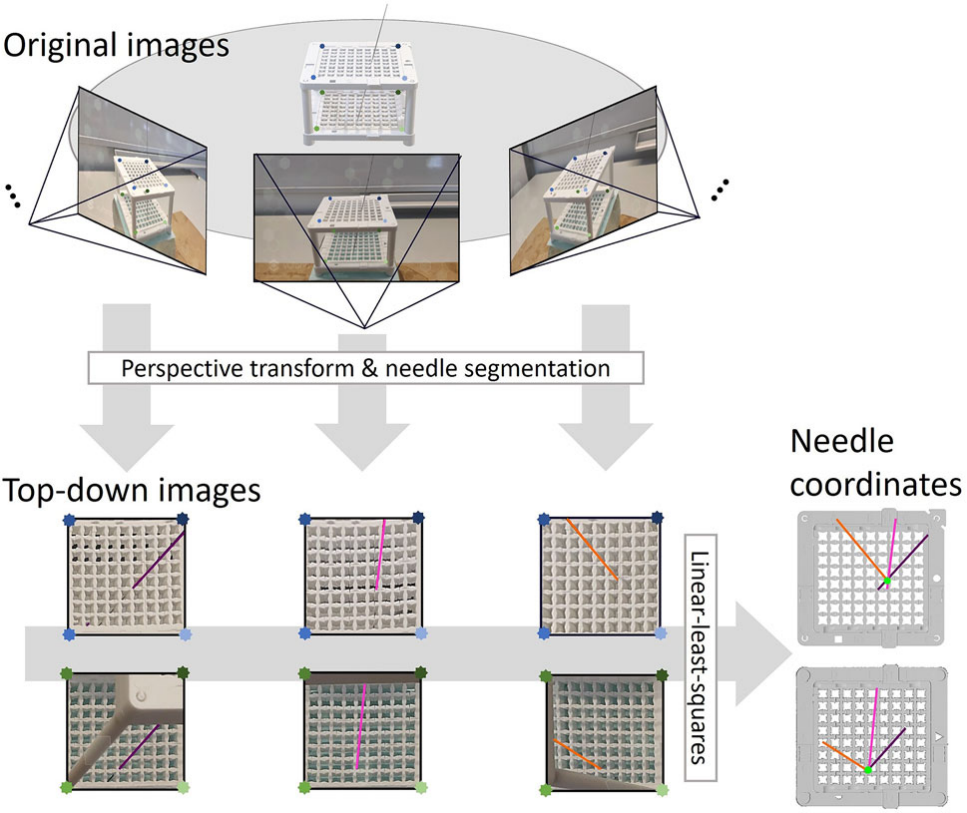
Key concept for identifying needle coordinates: Image frames are captured from different perspectives and transformed using the 6D-pose of the cube relative to the camera in order to obtain top-down view for both layers of the cube. On these, the needles are segmented with a convolutional neural network. For both layers, the intersection of the obtained lines is calculated using linear least squares, signifying the respective needle coordinate

Second, the correctness of the needle’s position is verified. Image frames from different perspectives of the tablet relative to the Navigation Cube are processed to identify the needle's coordinates on the cube's upper and lower grids (Fig. 3). If the needle is positioned correctly, the user receives positive feedback through a green checkmark and an affirmative sound. If the positioning is incorrect, a red cross and sound signal provide an immediate warning.

Third, the needle is inserted into the phantom. As the needle is being inserted, the rotation of the cube is monitored. As soon as the cube is moved more than 0.5 degrees, a holographic overlay of the cube gradually turns red, encouraging the user to maintain minimal rotation.

### Study design

**Fig. 4:**
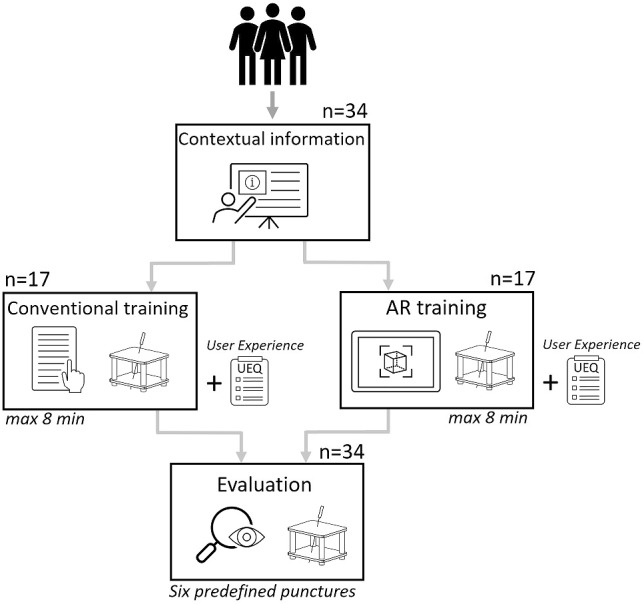
Illustration of the study flow: Participants receive contextual information before being randomly divided into two training groups. Following their training, they complete the user experience questionnaire for their respective training method. Subsequently all participants are being evaluated during six predefined punctures for the two possible user errors

This study included 34 medical students from different semesters (aged 19–43, 18 males, 16 females) (Fig. 4). A specific exclusion criterion was set to ensure that none of the participants had previous experience with the CNS. Ethical guidelines were adhered to throughout the study. Before the training, participants were given contextual information including purpose and possible applications of the CNS. For the training, they were randomly divided into two groups: One group received conventional written instructions for the application of the Navigation Cube, while the other group used the AR-training app. While training time was measured for each participant, the sessions were limited to a total duration of 8 min.

After training, all participants performed six punctures using the Navigation Cube on an evaluation setup. Thereby they were checked for errors leading to an inaccurate puncture, including (1) using incorrect coordinates and (2) rotating the cube.

#### Phantom

A tissue-mimicking phantom, designed to simulate soft human tissue and with dimensions of 80mm × 80mm × 60mm, was used for the training and evaluation procedures. The phantom was created using candle gel (Rayher GmbH, Germany) to represent tissue consistency. An elastically deformable consistency was deliberately selected to increase the difficulty for the participants to keep the cube from rotating. To emulate the resistance encountered during skin puncture, a paper mat was positioned over the gel surface.

#### Contextual information

To provide a comprehensive understanding of the CNS, a presentation was given to all participants before training which elucidated its fundamental concept.

#### AR-training

**Fig. 5:**
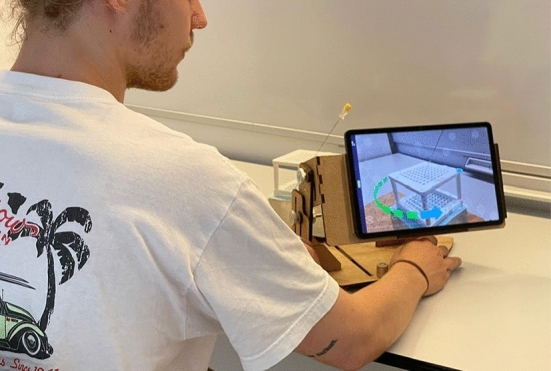
Participant performing AR-training on a phantom using the rotating holder

The AR-training group learned to puncture with the Navigation Cube by independently using the AR-app with a phantom and a needle. To enhance usability, a rotating holder was designed for the tablet (Fig. 5). This allows the tablet to easily pivot around the cube, freeing up the user's hands during the training process. Training time was limited to 8 min.

#### Conventional training

In the conventional training group, participants learned the usage Navigation Cube by individually reading a set of standard written instructions with written and visual information. They practiced using Navigation Cube with a phantom and a needle. Again, training time was limited to 8 min.

#### Evaluation

The evaluation protocol was standardized for both groups, ensuring that all 34 participants underwent identical procedures. To ensure a sufficiently robust dataset for assessing procedural user errors, each participant executed a series of six punctures using the Navigation Cube, with Cube Navigation System planning software displaying the targeted coordinates on a monitor. Mokry et al. also selected to perform six punctures in a similar study evaluating the accuracy when puncturing with Puncture Cubes [6].

**Fig. 6:**
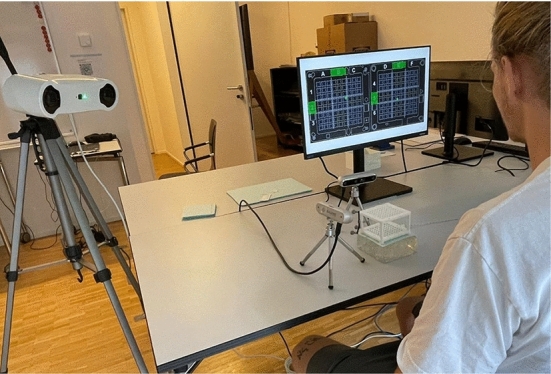
Evaluation setup with participant performing a predefined puncture on a phantom. Two perpendicularly placed cameras capture the procedure, whereas Atracsys system tracks the rotation of the Navigation Cube

With a deliberate setup (Fig. 6), the procedures were checked for the two sources of error: (1) using incorrect coordinates and (2) rotating the cube during the puncture. The correctness of the needle placement was manually verified using two RGB cameras, which captured footage of the needle as the puncture was performed. The needle was only considered to be correctly placed when the placement was correct for the upper and lower layer. Notably, this required hitting not only the correct holes but also the correct corners. A surgical tracking camera (Atracsys LLC, Puidoux, Switzerland) was used to record the rotation of the cube. The maximum change in angulation during each puncture was recorded. Equation (1) described by Du Q. Hu [16] was used to calculate cube rotation.

1 $$\uptheta ={\text{arccos}}\left(\frac{\left({\text{min}}\left({\text{trace}}\left({R}_{{\text{initial}}} * {{R}_{{\text{current}}}}^{{\text{T}}}\right), 3\right)- 1\right)}{2}\right)$$

where$$\begin{aligned}\\{R}_{{\text{initial}}}&:{\text{Rotation}}\; {\text{matrix}}\; {\text{of}}\; PC\; {\text{at}}\; {\text{starting}}\; \\ & \quad {\text{time}}\; {\text{of}}\; {\text{puncture}}\end{aligned}$$

$${R}_{{\text{current}}}:{\text{Rotation}} \; {\text{matrix}} \; {\text{of}} \; PC \; {\text{for}} \; {\text{current}} \; {\text{frame}}$$

### Statistical analysis

Statistical analyses were applied, focusing on the two errors possibly occurring during needle placement (using incorrect coordinates, rotating the cube). A binomial Generalized Linear Mixed-Effects Model was employed for analyzing binary outcomes, namely, the occurrence or non-occurrence of using wrong coordinates. In this model, the training group was treated as a fixed effect, while individual subjects were treated as random effects.

Conversely, a Linear Mixed-Effects Model was utilized for quantifying the continuous outcome extent of cube rotation. Again, the training group was the fixed effect, and the subjects were treated as random effects.

The Likelihood Ratio Test was conducted to ascertain whether the observed differences in error occurrence between the two groups were statistically significant. These analyses were conducted using the R environment for statistical computing (Version 4.3.1).

### User experience questionnaire

After training, all participants completed a user experience questionnaire (UEQ) [17] to compare the two groups' experiences. The questionnaire consists of 26 questions scaling the six categories: Attractiveness, Perspicuity, Dependability, Efficiency, Novelty, and Stimulation (Table 1). Participants rated their experience by selecting a point on the seven-point scale between contrasting adjectives (e.g., attractive/unattractive) that best represent their individual perception.

**Table 1:** UEQ scaling categories [17]

Attractiveness	What is the overall impression?
Perspicuity	Is it easy to get familiar with and is it easy to learn?
Efficiency	Can the tasks be solved quickly and without unnecessary effort?
Dependability	Is the system reliable and does the user feel in control of its handling?
Stimulation	Is it exciting and motivating?
Novelty	Is the product innovative and catchy?

Responses were converted to a scale from −3 to + 3, with scores above 0.8 considered positive, below 0.8 as negative, and scores in between as neutral. The categories were also benchmarked against a reference dataset and divided into five classes (Table 2) [18].

**Table 2:** UEQ benchmark classifying a product into 5 categories [18]

Rating	Benchmark comparison
Excellent	In the range of the 10% best results
Good	10% of the results in the benchmark dataset are better and 75% of the results are worse
Above average	25% of the results in the benchmark are better than the result for the evaluated product, and 50% of the results are worse
Below average	50% of the results in the benchmark are better than the result for the evaluated product, and 25% of the results are worse
Bad	In the range of the 25% worst results

## Results

### Training performance

Data from all 34 participants could be used for the evaluation. A total of 204 punctures were performed. The cube rotation measurements for 2 punctures had to be excluded due to incorrect recordings.

#### Training time

**Fig. 7:**
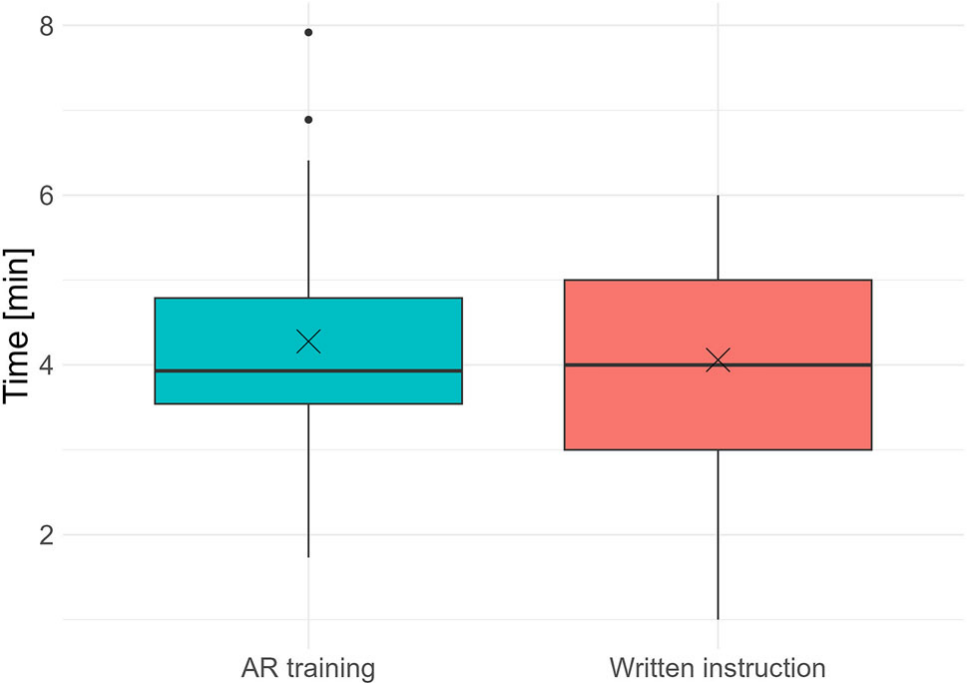
Distribution of training times for both groups

In Fig. 7, the boxplots depict the training durations for both groups. The average training time for each group was slightly over 4 min. No participant reached the maximum time of 8 min.

#### Correctness of needle coordinate

**Fig. 8:**
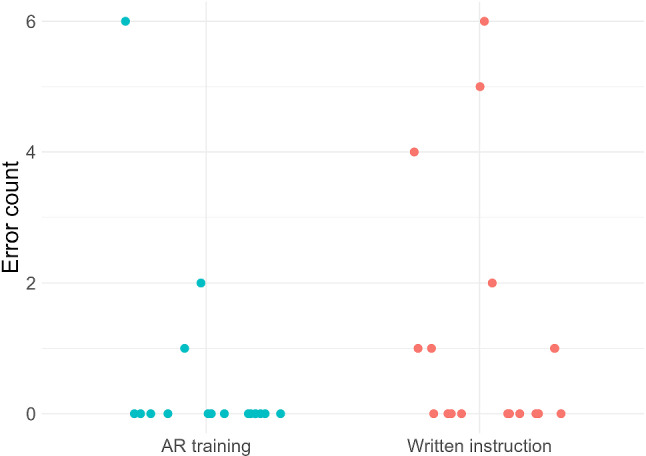
Distribution of error count in using incorrect coordinates for both groups

In evaluating the use of incorrect coordinates, it was found that in the AR-group, 3 out of 17 participants performed at least one of the six punctures incorrect. This resulted in 8.8% of all punctures being incorrect. Comparatively, in the group that was given conventional instructions, there was a higher error rate. When performing six evaluation punctures, 7 out of 17 participants in this group made one or more mistakes, leading to 19.6% of all punctures in this group being incorrect. The effect of the training group on the usage of correct and, respectively, incorrect coordinates was not significant (*p* > 0.05, binomial Generalized Linear Mixed-Effects Model). The scatter plot in Fig. 8 shows the distribution of the error count for each participant of both groups.

#### Cube rotation

**Fig. 9:**
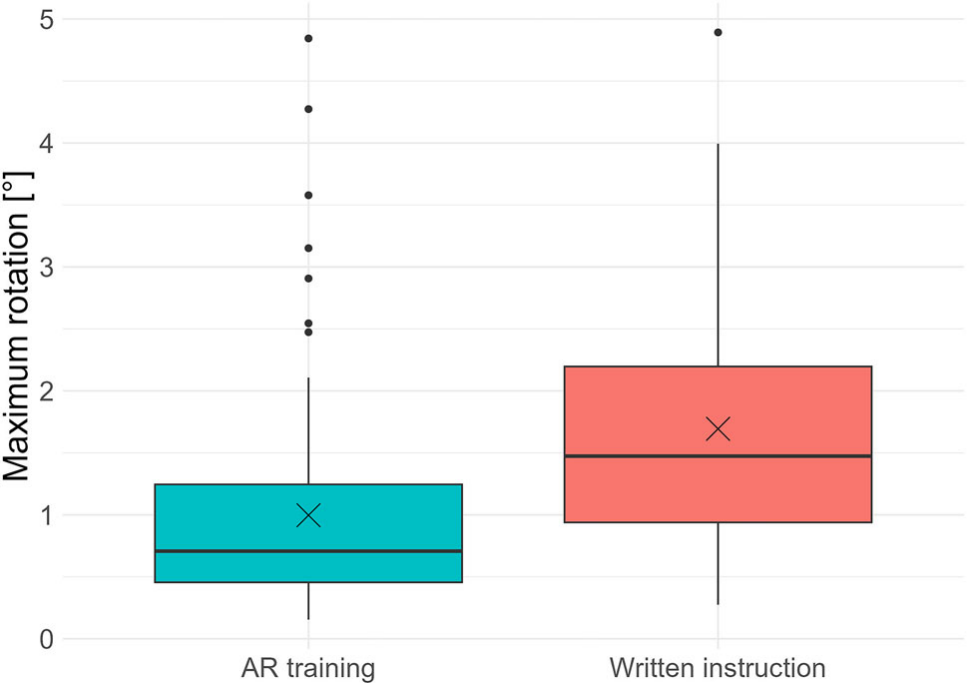
Distribution of maximal cube rotation for both groups

The boxplots in Fig. 9 show the maximum rotation of the cube during all performed evaluation punctures for both training groups. This was on average 1.0° for the AR-group and thus significantly lower (*p* < 0.01, Linear Mixed-Effect Model) than for the group trained with the conventional instructions at 1.7°.

### User experience

**Fig. 10:**
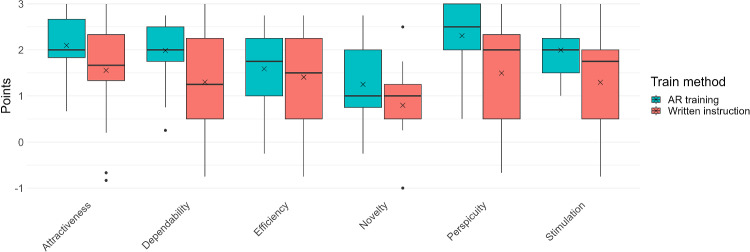
Distribution of UEQ scores for both groups and each category

The user experience of the AR-training was rated positively in all categories (> 0.8 points). When benchmarked against the UEQ reference dataset, attractiveness, perspicuity, dependability, and stimulation were considered “excellent”. Efficiency and novelty were rated as “good”. The conventional instruction also achieved positive scores for all categories. When benchmarked with the reference set, the conventional instructions achieve the rating “above average” in all categories. The AR-training was rated higher than the conventional instruction in all categories. Differences in the UEQ scores were significant for Attractiveness, Perspicuity, Dependability, Stimulation, and Novelty (*p* < 0.05, t test). Only for the category efficiency there was no significant difference. The boxplots in Fig. 10 show the distribution of the UEQ scores for both training groups.

## Discussion

In assessing both CNS training methods, this study highlights distinct advantages of AR over conventional written instructions. While both approaches proved effective, AR's potential to enhance the training effect and learning experience for needle placements emerged, attributable primarily to two factors: (1) the provision of immediate user feedback, and (2) the gradual, context-based delivery of information. These advantages underscore the promising role of AR in advancing safety and precision in percutaneous needle placements.

Both training methods required a comparable amount of time. Therefore, a direct comparison of the error occurrence between the two groups is valid. In assessing the first procedural error, the rate of using incorrect coordinates was halved for the AR-group. Nevertheless, no significant effect could be established. This is mainly due to low error occurrence and the high influence of the random effect subject. Moreover, the infrequent occurrence of errors implies that conventional instructions are already effective and that AR-instruction at minimum meets this standard. When examining the second procedural error, the AR-training group showed a significant decrease in cube rotation during the puncture process compared to the group trained with conventional instruction. The reduction of average cube rotation from 1.7° to 1.0° with AR-training compared to conventional instruction can be decisive for clinical applications. Laimer et al. defined a safety margin for CT-guided needle placement at 5 mm [19]. This safety margin translates to an angular deviation of 1.9° at a depth of 15 cm. An equivalent deviation from the planned needle path can result from cube movement of this magnitude. By analyzing the distribution of the two groups in Fig. 8, fewer instances of exceeding this limit in the AR-group than in the conventional instruction group were observed. The decrease in both procedural errors implies that AR training, by offering real-time feedback when errors occur, enables users to promptly adjust their actions, facilitating more effective learning. This highlights the first key advantage of AR over traditional written instructions.

User experience for the AR-training scored highly in all UEQ categories, four of six with the rating "excellent" suggesting users experienced the AR-training as engaging (Attractiveness, Stimulation), intuitive (Perspicuity), and reliable (Dependability). Accordingly, the scores were higher than for the conventional instructions. This positive feedback, superior to that for conventional instructions, can be attributed to the AR training's gradual, hands-on presentation of information, which likely reduces cognitive load and enhances user engagement. This underscores the second key advantage of AR training and indicates strong user acceptance, supporting its adoption as a preferred training method.

These results align with prior research investigating AR in medical training and further suggest the potential for broader adoption of AR-based training methods in medicine. The enhanced training performance and user experience justify the additional resources needed for AR-based methods, establishing it as a valid approach for CNS training in clinical practice. This study is the first to investigate training for navigation aids in percutaneous needle placements. Its findings are expected to be transferable to the training of other similar navigation devices.

The study accounts for some limitations: (1) The training and user evaluation were performed on a phantom. With this study design, the knowledge transfer from the simplified trainings setup to the complex clinical scenario cannot be evaluated. Therefore, implementing the evaluation in real-world scenario could be beneficial. (2) The study identified potential errors in CNS application but did not quantify their direct impact on puncture accuracy. Future research could compare puncture precision between the two groups to quantify the accuracy difference attributable to the training methods. (3) As the study was conducted on medical students, it is limited in its generalizability to working radiologists. For them, knowing the accuracy requirements of a puncture, it would be more obvious not to move the cube during puncture and to hit the coordinates exactly with the correct hole and corner. Therefore, subsequent studies could include practitioners, where a lower error rate would be expected. (4) The sample size in this study is not high enough to test the two groups for the correctness of the needle placements. Higher sizes would be beneficial for future studies. (5) The UEQ is intended for comparing two or more products within the same group of users. However, in this study, it was applied to compare user experiences between two groups. Although participants were randomly assigned to these groups from the same pool of medical students, this methodological choice introduces the possibility that random, inherent group differences could potentially skew the results in favor of one training method.

## Conclusion

This study suggests the potential of AR-training in enhancing training performance for navigation aids in needle placements. Compared to conventional written instructions, procedural errors were reduced with AR-training, while the training duration remained consistent. Furthermore, an enhanced user experience with the AR-training was reported by participants. Although the focus of this study was on the CNS training, the authors assume that these findings could be generalized to the training of other navigation aids for needle placements. The anticipated benefits of AR-training could lead to increased procedural safety, potentially resulting in improved treatment outcomes in percutaneous needle placements.

## References

[1] Li M, Seifabadi R, Long D, De Ruiter Q, Varble N, Hecht R, Negussie AH, Krishnasamy V, Xu S, Wood BJ (2020) Smartphone- versus smartglasses-based augmented reality (AR) for percutaneous needle interventions: system accuracy and feasibility study. Int J CARS 15:1921–1930. 10.1007/s11548-020-02235-7 10.1007/s11548-020-02235-7 PMC8985545 32734314

[2] Davrieux CF, Giménez ME, González CA, Ancel A, Guinin M, Fahrer B, Serra E, Kwak J-M, Marescaux J, Hostettler A (2020) Mixed reality navigation system for ultrasound-guided percutaneous punctures: a pre-clinical evaluation. Surg Endosc 34:226–230. 10.1007/s00464-019-06755-5 30911919 10.1007/s00464-019-06755-5

[3] Chehab M, Brinjikji W, Copelan A, Venkatesan A (2015) Navigational tools for interventional radiology and interventional oncology applications. Semin intervent Radiol 32:416–427. 10.1055/s-0035-1564705 26622105 10.1055/s-0035-1564705 PMC4640922

[4] Durand P, Moreau-Gaudry A, Silvent A-S, Frandon J, Chipon E, Médici M, Bricault I (2017) Computer assisted electromagnetic navigation improves accuracy in computed tomography guided interventions: a prospective randomized clinical trial. PLoS ONE 12:e0173751. 10.1371/journal.pone.0173751 28296957 10.1371/journal.pone.0173751 PMC5351986

[5] Scharll Y, Mitteregger A, Laimer G, Schwabl C, Schullian P, Bale R (2022) Comparison of a robotic and patient-mounted device for CT-guided needle placement: a phantom study. JCM 11:3746. 10.3390/jcm11133746 35807029 10.3390/jcm11133746 PMC9267795

[6] Mokry A, Willmitzer F, Hostettler R, Richter H, Kircher P, Kneissl S, Wetzel S (2019) Evaluation of a novel, patient-mounted system for CT-guided needle navigation—an ex vivo study. Neuroradiology 61:55–61. 10.1007/s00234-018-2107-0 30506482 10.1007/s00234-018-2107-0

[7] Barsom EZ, Graafland M, Schijven MP (2016) Systematic review on the effectiveness of augmented reality applications in medical training. Surg Endosc 30:4174–4183. 10.1007/s00464-016-4800-6 26905573 10.1007/s00464-016-4800-6 PMC5009168

[8] Tang KS, Cheng DL, Mi E, Greenberg PB (2020) Augmented reality in medical education: a systematic review. Can Med Educ J 11:e81–e96. 10.3683/cmej.61705 32215146 10.36834/cmej.61705 PMC7082471

[9] Lia H, Paulin G, Yi N, Haq H, Emmanuel S, Ludig K, Keri Z, Lasso A, Fichtinger G, Yeo CT, Andrews J (2018) HoloLens in suturing training. In: Webster RJ, Fei B (eds) Medical imaging 2018: image-guided procedures, robotic interventions, and modeling. SPIE, Houston, United States, p 69

[10] Azimi E, Winkler A, Tucker E, Qian L, Doswell J, Navab N, Kazanzides P (2018) Can mixed-reality improve the training of medical procedures? In: 2018 40th annual international conference of the IEEE engineering in medicine and biology society (EMBC). IEEE, Honolulu, HI, pp 4065–4068 10.1109/EMBC.2018.8513387 30441249

[11] Wolf J, Wolfer V, Halbe M, Maisano F, Lohmeyer Q, Meboldt M (2021) Comparing the effectiveness of augmented reality-based and conventional instructions during single ECMO cannulation training. Int J CARS 16:1171–1180. 10.1007/s11548-021-02408-y 10.1007/s11548-021-02408-y PMC8260416 34023976

[12] Ungi T, Sargent D, Moult E, Lasso A, Pinter C, McGraw RC, Fichtinger G (2012) Perk tutor: an open-source training platform for ultrasound-guided needle insertions. IEEE Trans Biomed Eng 59:3475–3481. 10.1109/TBME.2012.2219307 23008243 10.1109/TBME.2012.2219307

[13] Holden MS, Xia S, Lia H, Keri Z, Bell C, Patterson L, Ungi T, Fichtinger G (2019) Machine learning methods for automated technical skills assessment with instructional feedback in ultrasound-guided interventions. Int J CARS 14:1993–2003. 10.1007/s11548-019-01977-3 10.1007/s11548-019-01977-3 31006107

[14] Hayasaka T, Kawano K, Onodera Y, Suzuki H, Nakane M, Kanoto M, Kawamae K (2023) Comparison of accuracy between augmented reality/mixed reality techniques and conventional techniques for epidural anesthesia using a practice phantom model kit. BMC Anesthesiol 23:171. 10.1186/s12871-023-02133-w 37210521 10.1186/s12871-023-02133-w PMC10199582

[15] Krammer L, Hostettler R, Wetzel S (2023) Evaluation of the access cube patient-mounted navigation system for CT-guided percutaneous needle placement–a phantom study. J Vascular Interv Radiol. 10.1016/j.jvir.2023.06.036 10.1016/j.jvir.2023.06.036 37406773

[16] Huynh DQ (2009) Metrics for 3D rotations: comparison and analysis. J Math Imaging Vis 35:155–164. 10.1007/s10851-009-0161-2

[17] Laugwitz B, Held T, Schrepp M (2008) Construction and evaluation of a user experience questionnaire. In: Holzinger A (ed) HCI and usability for education and work. Springer, Berlin Heidelberg, Berlin, Heidelberg, pp 63–76

[18] Schrepp M (2015) User experience questionnaire handbook. All you need to know to apply the UEQ successfully in your project. 10.13140/RG.2.1.2815.0245

[19] Laimer G, Jaschke N, Schullian P, Putzer D, Eberle G, Solbiati M, Solbiati L, Goldberg SN, Bale R (2021) Volumetric assessment of the periablational safety margin after thermal ablation of colorectal liver metastases. Eur Radiol 31:6489–6499. 10.1007/s00330-020-07579-x 33447860 10.1007/s00330-020-07579-x PMC8379110

